# Mineral and bone disorder and longterm survival in a chronic kidney disease grade 3b-4cohort

**DOI:** 10.1080/0886022X.2022.2107543

**Published:** 2022-08-10

**Authors:** Pablo Rios, Ricardo Silvariño, Laura Sola, Alejandro Ferreiro, Verónica Lamadrid, Laura Fajardo, Liliana Gadola

**Affiliations:** a Comisión Asesora Programa de Salud Renal, Fondo Nacional de Recursos, Montevideo, Uruguay; b Facultad de Medicina, Universidad de la República, Montevideo, Uruguay; c Sociedad Uruguaya de Nefrología, Montevideo, Uruguay

**Keywords:** Calcium, chronic kidney disease 3b-4, mineral metabolism, parathyroid hormone, phosphate, survival analysis

## Abstract

Mineral and bone disorder biomarkers ‘normal ranges’ are controversial. The aim of the study was to evaluate the association between serum calcium (Ca), phosphate (P), intact parathyroid hormone (iPTH), and 25(OH) vitamin D levels and mortality risk, in a chronic kidney disease (CKD) grade (G) 3b-4 cohort. The Uruguayan National Renal Healthcare Program (NRHP-UY) CKD patients’ cohort, included between 1 October 2004 and 1 March 2020 and followed-up until 1 March 2021, was analyzed with the Ethics Committee approval. A total of 6473 patients were analyzed: 56% men, median age 73 (65–79) years, 55% on CKD G3b. At the end of the follow-up, 2459 (37.7%) patients had died (6.4/100 patient–year). There were iPTH data on 2013 patients (younger, with lower estimated glomerular filtration rate (eGFR) and lesser comorbidities). By bivariate Cox analysis the lowest death risk was observed with mean Ca between 9.01 and 10.25 mg/dl, P between 2.76 and 4.0 mg/dl, iPTH ≤ 105 pg/ml, and 25(OH) vitamin D >10 ng/ml. The multivariate Cox regression mortality risk adjusted to age, sex, CKD etiology, diabetes, smoking, cardiovascular comorbidity, blood pressure, proteinuria, eGFR, renin-angiotensin system blockers and vitamin D treatments, serum Ca, P, iPTH, and 25(OH) vitamin D (*n* = 964) showed that a higher mortality risk was associated with *p* > 4.00 mg/dl (HR 1.668, CI 95%: 1.201–2.317), iPTH >105 pg/ml (HR 1.386, CI 95%: 1.012–1.989), and 25(OH) vitamin D ≤ 10 ng/ml (HR 1.958, CI 95%: 1.238–3.098) and a lower mortality risk with 1,25(OH)_2_ vitamin D treatment (HR 0.639, CI 95%: 0.451–0.906). These data may contribute to the precise G3b-4 CKD-MBD biomarkers levels definition.

## Introduction

Chronic kidney disease and related mineral and bone disorders (CKD-MBD) are very frequent and are associated with bone disease, vascular calcifications and risk of all-cause and cardiovascular death [1–8]. The international Kidney Disease Improving Global Outcomes (KDIGO) guidelines 2017 for CKD-MBD suggest (2C) maintaining serum calcium, phosphate, and parathyroid hormone (PTH) within normal ranges [9]. Nevertheless, the authors highlighted that there are no data supporting that this is of benefit to CKD Grade (G)3a–G4 patients. Recently, two large multicentric studies have been published on patients on dialysis (COSMOS study) [10,11] and on CKD G3–4 [12,13] that evaluated the association between CKD-MBD serum biomarkers and mortality risk. They observed a nonlinear association between serum calcium, phosphate, and PTH and mortality risk on a follow-up of 36 months.

Since 2004 a National Renal Healthcare Program [14,15] was developed in Uruguay (NRHP-UY) to promote education on kidney diseases, to improve prevention and early CKD diagnosis and treatment (http://www.fnr.gub.uy/home_psaludrenal). It has developed a national registry that included CKD G1-G5 nondialysis patients that enables to epidemiological analysis.

The aim of the present study was to evaluate the association among serum calcium, phosphate, PTH, and 25(OH) vitamin D levels and mortality risk, in a large CKD G3b-4 cohort with long follow-up time.

## Materials and methods

The NRHP-UY Registry includes patients with CKD diagnosis, defined as an estimated glomerular filtration rate (eGFR) less than 60 mL/min/1.73 m^2^ and/or proteinuria ≥150 mg/day (or albuminuria ≥30 mg/day in diabetics) for at least 3 months, who are voluntarily included in the CKD Registry (http://www.fnr.gub.uy). Therapeutic goals are clearly defined [14] and monitored (http://www.fnr.gub.uy/home_psaludrenal). The 50 NRHP-UY Units cover up to 74.8% of the country’s population. The objectives, implementation, and the preliminary results of the NRHP-UY have been described previously [15–17].

In this study, longitudinal data were analyzed for a CKD adult patient cohort, prospectively admitted to the NRHP-UY registry between 1 October 2004 and 1 March 2020, followed-up until 1 March 2021.

**Figure 1.**
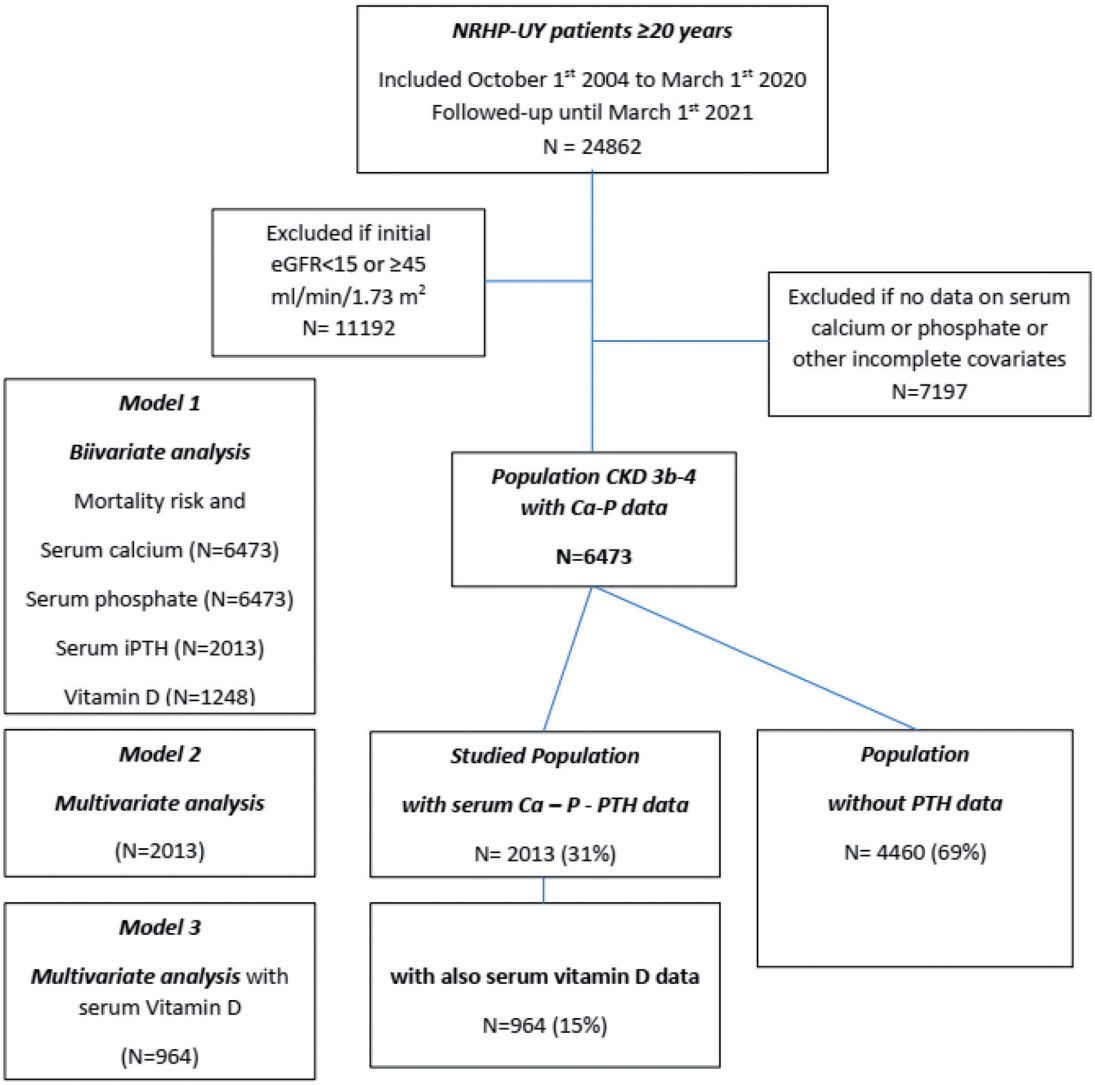
Algorithm of the Uruguayan National Renal Healthcare Program (NRHP-UY) population and the distribution of the studied groups. eGFR: estimated glomerular filtration rate; Ca: calcium; P: phosphate; iPTH: intact parathyroid hormone; vitamin D : 25(OH) vitamin D.

The cohort inclusion criteria were: (a) meeting the admission criteria for the NRHP-UY, (b) being 20 years or older, and (c) being on CKD G3b-G4 at inclusion. The exclusion criteria were lacking data of serum calcium or phosphate on evolution (Figure 1).

Age, sex, CKD etiology, comorbidities (diabetes, hypertension, smoking, ischemic heart disease, chronic heart failure, peripheral arteriopathy, and/or stroke), and laboratory and clinic data were prospectively registered upon admission to the NRHP-UY and at every clinic visit: body mass index (BMI), systolic and diastolic blood pressure (SBP/DBP), proteinuria, serum creatinine, eGFR, serum calcium, serum phosphate, serum intact (i) PTH, and treatments with renin–angiotensin–aldosterone system blockers (RASB), 25(OH), and 1,25(OH)_2_ vitamin D at inclusion and/or on follow-up. Estimated glomerular filtration rate was calculated per CKD-EPI equation [18] (as creatinine measures are standardized to isotope dilution mass spectrometry traceable methods all-over the country) and CKD stages were defined by K-DIGO 2012 [19]. Proteinuria was measured following national [20] and international criteria [19], informed as proteinuria/creatininuria, albuminuria/creatininura, or 24 hs proteinuria. The serum calcium, phosphate, iPTH, and 25(OH) vitamin D data were those registered by each nephrological team, as measured at the local laboratory departments by the standardized methods used routinely, and their mean levels were calculated as the mean of all data on each patient’s records. Death and kidney replacement treatment (KRT) initiation were actively monitored until 1 March 2021. All deaths in the country are recorded in the Death Registry of the Ministry of Health, and the national mandatory Registry of Chronic KRT includes every person receiving chronic dialysis or renal transplantation across the country. If a patient was not admitted to KRT or died (as for the mentioned mandatory national registries), that patient was deemed alive by 1 March 2021.

The primary outcome measured was all-cause death.

### Statistical analysis

For the descriptive analysis, data are presented as summary measures (median and interquartile range, percentage, and confidence interval with 95% dispersion). Tests adjusted to variable nature and distribution were used for the statistical inference analysis (Mann–Whitney, Chi^2^, Wilcoxon tests). Risk estimation was conducted by calculating the hazard ratio (HR) by multivariate Cox’s regression, with the corresponding 95% confidence interval (CI), adjusted to covariates.

**Figure 2.**
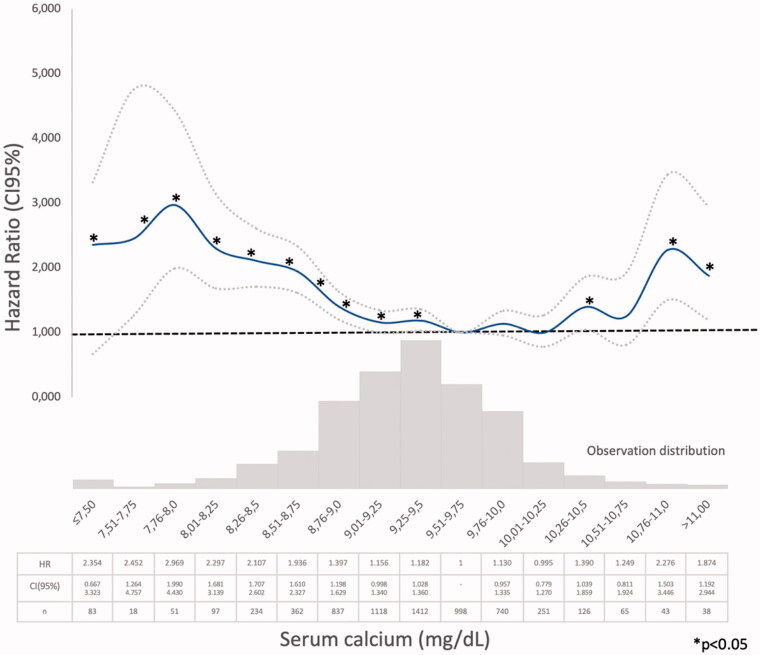
Hazard ratio (HR) and 95% confidence interval (CI) for the death risk associated with mean serum calcium ranges (Model 1).

**Figure 3.**
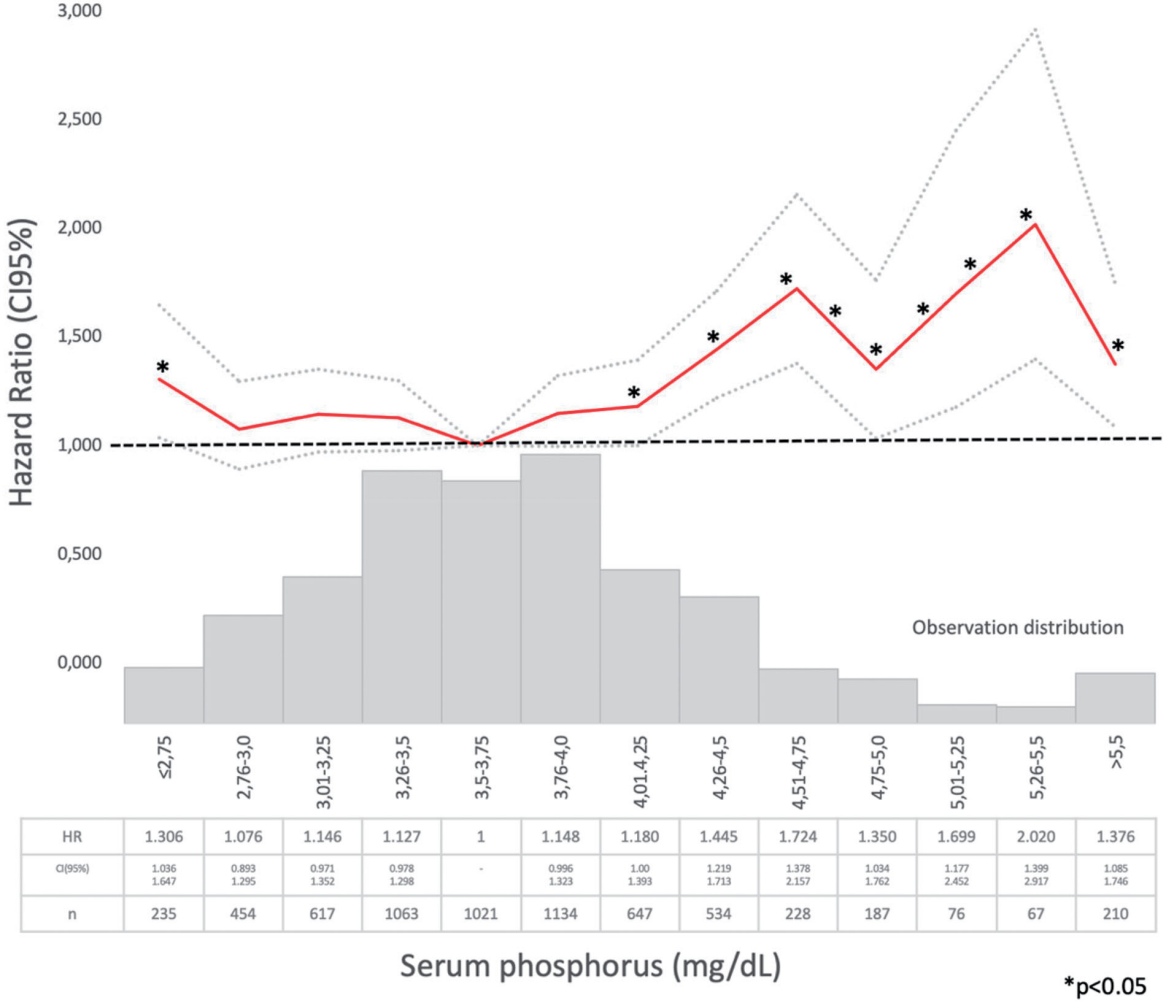
Hazard ratio (HR) and 95% confidence interval (CI) for the death risk associated with mean serum phosphate ranges (Model 1).

**Figure 4.**
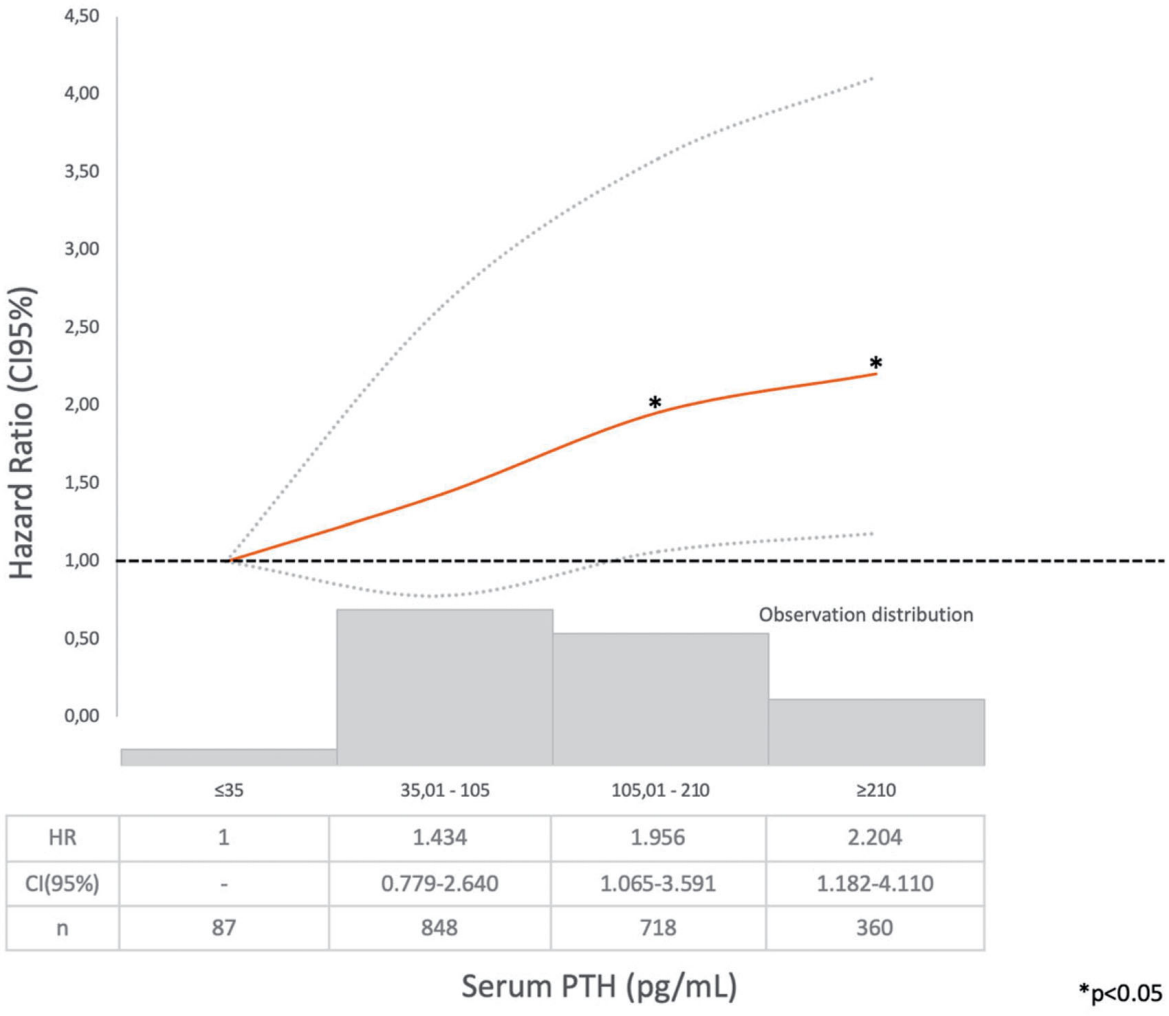
Hazard ratio (HR) and 95% confidence interval (CI) for the death risk associated with mean serum parathyroid hormone (iPTH) ranges (Model 1).

**Figure 5.**
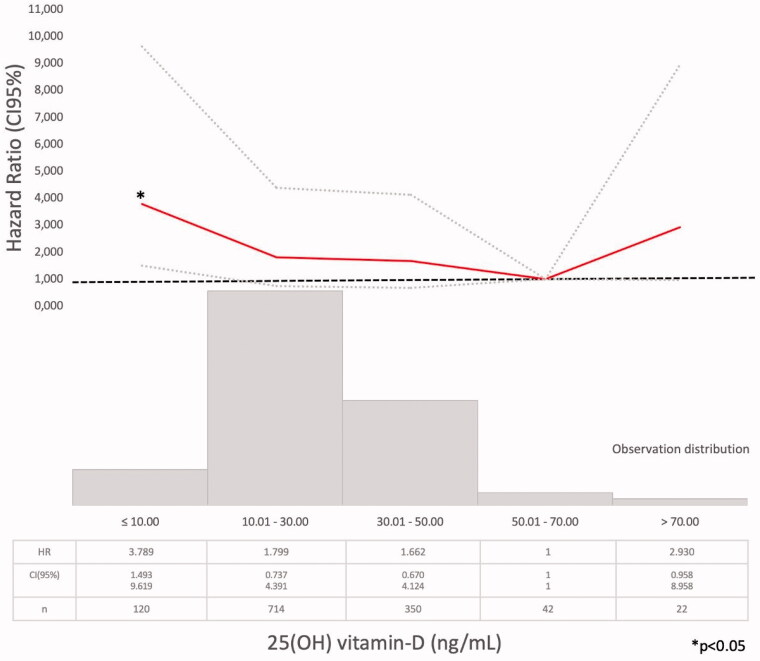
Hazard ratio (HR) and 95% confidence interval (CI) for the death risk associated with mean serum 25(OH) vitamin D ranges (Model 1).

Three different models were used to assess mortality risk: Model 1: bivariate Cox regression survival analysis of each of the MBD biomarkers (mean serum calcium, phosphate, iPTH, and 25(OH) vitamin D) classified in small ranges categories, separately, in order to identify the ranges associated with the lower mortality risk (HR ≤1.2 and/or *p* > .05) vs. the lowest risk range observed in each one (Figures 2–5). Afterwards, the serum values were categorized as below, within, and above the lower mortality risk ranges, if suitable (Supplementary Tables 1–4). Model 2 (*n* = 2013): multivariate Cox regression analysis adjusted for: age, sex, CKD etiology, diabetes, smoking, cardiovascular comorbidity, blood pressure, proteinuria, eGFR, BSRA, treatment with 25(OH), and 1,25(OH)_2_ vitamin D and the three BMD biomarkers (serum calcium, phosphate, and iPTH categorized as previously described) (Supplementary Table 5) and Model 3 (*n* = 962) adjusted also for serum 25(OH) vitamin D categorized (Supplementary Table 6). In every case, the null hypothesis was rejected at *p* < .05 or with overlapping 95% confidence intervals. IBM SPSS 15.0, software was used for the analysis.

### Ethics

Patients included in the NRHP-UY Registry signed an informed consent. Pursuant to Law No. 18331 (*Habeas Data*) and as to maintain confidentiality, no identifying sensitive data were included in the databases. The systematic analysis of data from the NRHP-UY was approved by the Ethics Committee of the Faculty of Medicine of the Universidad de la República (25 September 2006) (File No. 071140-002077-06) and this specific analysis was registered at the Ministry of Health (No. 4523431.) and approved by the Ethics Committee of the Hospital de Clínicas, Universidad de la República (File 91-21_20211213_0001). This study adhered to the guidelines by the STROBE group for cohort observational studies [21].

## Results

### Population

Since 1 October 2004 to 1 March 2020, 24,862 patients were admitted to the NRHP-UY, and 6473 accomplished inclusion criteria (Figure 1). They were 56% men with a median age of 73 (65–79) years. On admission to NRHP-UY, 3558 (55%) were on CKD G3b and 2915 (45%) on CKD G4, proteinuria was absent or below 300 mg/day in 5272 (81.5%), baseline systolic blood pressure was less than 140 mmHg in 58.7%, and diastolic blood pressure was less than 90 mmHg in 82.7%. The most frequent CKD etiology were vascular (52.3%), diabetic (12.2%), tubulointerstitial nephropathies (5.6%), and glomerulopathies (2.8%). They had frequent vascular risk factors and comorbidities as diabetes (37.8%), overweight-obesity (75.9%), cardiovascular comorbidities (34.6%), and/or smoking (5.9%) (Table 1).

**Table 1.** Baseline data.

	Global	PTH-data group	No PTH-data group	*p*
Number	6473	2013	4460	
Age (years) (median, pc 25–75)	73.7 (65.9–79.6)	71.5 (63.1–77.8)	74.6 (67.0–80.3)	<.001^a^
Sex (male), *n* (%)	3628 (56.0)	1141 (56.7)	2487 (55.8)	.254^a^
CKD etiology				
Vascular, *n* (%)	3388 (52.3)	973 (48.3)	2415 (54.1)	<.001^b^
Diabetic, *n* (%)	789 (12.2)	204 (10.1)	585 (13.1)	
Tubulo-interstitial/Obstructive, *n* (%)	364 (5.6)	105 (5.2)	259 (5.8)	
Glomerulopathies, *n* (%)	181 (2.8)	67 (3.3)	114 (2.6)	
CKD Grade 3b	3558 (55.0)	951 (47.2)	2607 (58.5)	<.001^b^
CKD Grade 4	2915 (45.0)	1062 (52.8)	1853 (41.5)	
Comorbidities				
Diabetes, *n* (%)	2447 (37.8)	740 (36.8)	1707 (38.3)	.128^b^
Smoking, *n* (%)	382 (5.9)	105 (5.2)	277 (6.2)	.124^b^
Cardiovascular events (1 or more)	2242 (34.6)	618 (30.7)	1624 (36.4)	<.001^b^
SB*p* < 120 mmHg, *n* (%)	1148 (17.7)	372 (18.5)	776 (17.4)	.573^b^
120–139 mmHg, *n* (%)	2654 (41.0)	826 (41.0)	1828 (41.0)	
140–159 mmHg, *n* (%)	1845 (28.5)	554 (27.5)	1291 (28.9)	
≥160 mmHg, *n* (%)	826 (12.8)	261 (13.0)	565 (12.7)	
DB*p* < 80 mmHg), *n* (%)	3231 (49.9)	1030 (51.2)	2201 (49.3)	.469^b^
80–89 mmHg, *n* (%)	2125 (32.8)	645 (32)	1480 (33.2)	
90–99 mmHg, *n* (%)	783 (12.1)	231 (11.5)	552 (12.4)	
≥100 mmHg, *n* (%)	334 (5.2)	107 (5.3)	227 (5.1)	
BMI (kg/m^2^) (<21*)*, *n* (%)	229 (4.6)	63 (3.9)	166 (4.9)	.272^b^
BMI (kg/m^2^) [21–25], *n* (%)	948 (18.9)	289 (18.0)	659 (19.3)	
BMI (kg/m^2^) (>25*)*, *n* (%)	2590 (75.9)	1253 (78.1)	3843 (76.6)	
eGFR (ml/min/1.73 m^2^) (median, pc 25–75)	31.5 (23.9–37.9)	29.3 (22.5–36.2)	32.4 (24.9–38.5)	<.001^a^
Proteinuria (data *n* = 6228)				.312^b^
No	4748 (73.4)	1503 (74.7)	3245 (72.8)	
<300 mg/d (o PCR < 300 mg/g), *n* (%)	524 (8.1)	162 (8.0)	362 (8.1)	
300–1000 mg/d (o PCR 300–1000 mg/g), *n* (%)	726 (11.2)	215 (10.7)	511 (11.5)	
≥1000 mg/d (o PC*R* < 1000 mg/g), *n* (%)	475 (7.3)	133 (6.6)	342 (7.7)	
BSRA, *n* (%)	4665 (72.1)	1446 (71.8)	3219 (72.2)	.399^b^

SBP: systolic blood pressure; DBP: diastolic blood pressure; BMI: body mass index; eGFR: estimated glomerular filtration rate.

^a^Mann–Whitney test.

^b^Chi^2^ test.

There were serum iPTH data on 2013 patients’ records, so the bivariate iPTH (Model 1) and Model 2 analysis could be performed on these patients, that was defined as the PTH-data Group and its baseline characteristics were compared with the No PTH-data Group. The PTH-data Group included younger patients, with lower eGFR and less cardiovascular comorbidities (Table 2).

**Table 2.** CKD-MBD data.

	Global	PTH-data	No PTH-data	*p*
Patients (*n*)	6473	2013	4660	
Frequencies KDIGO ranges				
Serum calcium^a^				
≤8.40 mg/dl, *n* (%)	325 (5.0)	62 (3.1)	263 (5.9)	<.001^b^
8.41–10.00 mg/dl, *n* (%)	5625 (86.9)	1805 (89.7)	3820 (85.6)	
>10.00 mg/dl, *n* (%)	523 (8.1)	146 (7.3)	377 (8.5)	
Serum phosphate^a^ x				
≤8.40 mg/dl, *n* (%)	537 (8.3)	149 (7.4)	388 (8.7)	<.001^b^
3.01–4.50 mg/dl, *n* (%)	5077 (78.4)	1667 (82.8)	3410 (76.5)	
>4.50 mg/dl, *n* (%)	859 (13.3)	197 (9.8)	662 (14.8)	
iPTH (data *n* = 1943)^a^				
≤35.0 pg/ml, *n* (%)	–	87 (4.3)	**–**	
35.1–70.0 pg/ml, *n* (%)	–	397 (19.7)	**–**	
70.1–105.0 pg/ml, *n* (%)	–	451 (22.4)	**–**	
>105.0 pg/ml, *n* (%)	–	1078 (53.5)	**–**	
Frequencies Model 1 mortality risk ranges				
Serum calcium^a^ ≤9.00 mg/dl, *n* (%)	1682 (26.0)	430 (21.4)	1252 (28.1)	<.001^b^
9.01–10.25 mg/dl, *n* (%)	4519 (69.8)	1508 (74.9)	3011 (67.5)	
>10.25 mg/dl, *n* (%)	272 (4.2)	75 (3.7)	197 (4.4)	
Serum phosphate^a^ ≤2.75 mg/dl, *n* (%)	235 (3.6)	61 (3.0)	174 (3.9)	<.001^b^
2.76–4.00 mg/dl, *n* (%)	4289 (66.3)	1401 (69.6)	2888 (64.8)	
>4.0 mg/dl, *n* (%)	1949 (30.1)	551 (27.4)	1399 (31.3)	
Serum 25(OH) vitamin D (data *n* = 1248)^a^ ≤10 ng/ml, *n* (%)	120 (9.6)	85 (8.8)	35 (12.3)	.078^b^
>10 ng/ml, *n* (%)	1128 (90.4)	879 (91.2)	249 (87.7)	
iPTH (data *n* = 2013)^a^ ≤105 pg/ml, *n* (%)	–	935 (46.4)	–	
>105 pg/ml, *n* (%)	–	1078 (53.6)	–	
Serum levels				
Serum calcium (mg/dl) (median, pc 25–75)	9.33 (9.00–9.66)	9.36 (9.05–9.64)	9.30 (9.00–9.67)	.023^c^
Serum phosphate (mg/dl) (median, pc 25–75)	3.70 (3.34–4.13)	3.70 (3.37–4.05)	3.70 (3.30–4.20)	.214^c^
Serum iPTH (pg/ml) (median, pc 25–75)	–	112 (72–176)	–	
Serum 25(OH) vitamin D (ng/ml) (data *n* = 1248) (median, pc 25–75)	25.5 (17.0–33.0)	25.7 (17.2–33.0)	25.1 (15.9–33.5)	.645^c^
Treated 25(OH) vitamin D^d^ (data *n* = 5215), *n* (%)	1294 (24.8)	919 (45.6)	375 (11.7)	<.001^b^
Treated1, 25(OH)_2_ vitamin D^e^ (data *n* = 5215), *n* (%)	710 (13.6)	512 (25.4)	198 (6.2)	<.001^b^

iPTH: intact parathyroid hormone.

^a^Mean values.

^b^Chi^2^ test.

^c^Mann–Whitney test.

^d^Treated 25(OH) vitamin D: 20.6% on CKD G3b vs. 30.2% on CKD G4 (Chi^2^ test *p* < .05).

^e^Treated 1,25(OH)_2_ vitamin D: 10.2% on CKD G3b vs. 18.0% on CKD G4 (Chi^2^ test *p* < .05).

According to KDIGO 2009 and SEN 2011 CKD-MBD Guidelines [7,22] biomarkers were on the target ranges on (Table 2): 86.9% for mean serum calcium (8.4–10.0 mg/dl), 78.4% for mean serum phosphate (3.0–4.5 mg/dl), 19.7% for mean iPTH (35–70 pg/ml), and 22.4% for mean iPTH (70–105 pg/ml). On CKD G3b group 254 (27.9%) had mean iPTH (35–70 pg/ml) and on CKD G4 group 203 (20.1%) had mean iPTH (70–105 pg/ml).

Serum 25(OH) vitamin D was measured in1248 patients (25.5 (17.0–33.0) ng/ml), without differences between PTH groups. There were 964 in the PTH-data group, so Model 3 was applied only to them. From them, 85 patients (8.8%) had mean serum 25(OH) vitamin D ≤ 10 ng/ml. There were data on vitamin D treatment in 5215 patients, 24.8% received 25(OH) vitamin D (20.6% on CKD G3b vs. 30.2% on CKD G4) and 13.6% received 1,25(OH)_2_ vitamin D (10.2% on CKD G3b vs. 18.0% on CKD G4) significantly more on CKD G4 and on the PTH-data Group (Chi^2^ *p* < .05) (Table 2).

Median follow-up time was 65.5 (33.2–102.1) months, significantly longer on the PTH-data group 81.5 (47.9–113.6) vs. No PTH-data group 59.0 (28.2–95.1) (Table 2). At the end of follow-up, 2459 (37.7%) patients had died with incidence rate 6.4/100 patient–year (3.4 in the PTH-data group) (Table 2).

### Mortality risk analysis

Model 1 (Supplementary Tables 1–4). The Cox regression bivariate analysis showed that the lowest mortality risks, according to the proposed methodology, were observed within the ranges of serum calcium 9.01–10.25 mg/dl (Figure 2) and serum phosphate 2.76–4.0 mg/dl (Figure 3). Serum iPTH data were available (as referred) in 2013 patients. In this studied group, the bivariate Cox regression (subdivided in four ranges, with reference PTH ≤35 pg/ml) showed all HR >1.2 (Figure 4). So iPTH ranges were re-adjusted and the serum iPTH range associated with a significantly higher mortality risk was iPTH ≥105 pg/ml. Serum 25(OH) vitamin D data were available in 1248 patients and in this group, the bivariate Cox regression analysis showed that only serum 25(OH) vitamin D levels ≤10 ng/ml were significantly associated with a higher mortality risk (Figure 5).

Model 2 (Supplementary Tables 4 and 5). The multivariate Cox regression mortality risk adjusted to age, sex, CKD etiology, diabetes, smoking, cardiovascular comorbidity, blood pressure, proteinuria, eGFR, BSRA, 25(OH), and 1,25(OH) vitamin D treatment and MBD biomarkers (calcium, phosphate, and iPTH) showed that a higher mortality risk was associated with age (HR 1.080, CI 95%: 1.067–1.094), diabetes (HR 1.510, CI 95%: 1.219–1.851), cardiovascular comorbidity (HR 1.328, CI 95%: 1.090–1.619), proteinuria (HR 1.688, CI 95%: 1.249–2.280), serum calcium ≤9.00 mg/dl (HR 1.391, CI 95%: 1.127–1.707, *p* = .002), serum phosphate >4.00 mg/dl (HR 1.387, CI 95%: 1.11–1.720, *p* = .003), and iPTH >105 pg/ml (HR 1.275, CI 95%: 1.049–1.550, *p* = .015) and a lower mortality risk was independently associated with RASB treatment (HR 0.749, CI 95%: 0.608–0.922) and 25(OH) vitamin D treatment (HR 0.704, CI 95%: 0.575–0.861).

Model 3 (Supplementary Tables 5 and 6). The multivariate Cox regression mortality risk adjusted to age, sex, CKD etiology, diabetes, smoking, cardiovascular comorbidity, blood pressure, proteinuria, eGFR, BSRA, 25(OH), and 1,25(OH) vitamin D treatment and MBD biomarkers: calcium, phosphate, iPTH, and serum 25(OH) vitamin D levels showed that a higher mortality risk was associated with serum phosphate >4.00 mg/dl (HR 1.668, CI 95%: 1.201–2.317), iPTH >105 pg/ml (HR 1.386, CI 95%: 1.012–1.989), and 25(OH) vitamin D ≤ 10 ng/ml (HR 1.958, CI 95%: 1.238–3.098) and a lower mortality risk with 1,25(OH)_2_ vitamin D treatment (HR 0.639, CI 95%: 0.451–0.906).

## Discussion

In a large CKD G3b-4 cohort, with a long follow-up time, it was observed, in the bivariate analysis, that the lowest mortality risks were observed within the ranges of serum calcium 9.01–10.25 mg/dl (Figure 2) and serum phosphate 2.76–4.0 mg/dl (Figure 3). In the multivariate analysis, performed in the PTH-data group, a higher adjusted mortality risk was associated with low (≤9.00 mg/dl) serum calcium levels (in Model 2 not adjusted to vitamin D level), high serum phosphate (>4.00 mg/dl), and iPTH >105 pg/ml. Mean serum calcium, phosphate, PTH, and 25(OH) vitamin D levels were analyzed, as the available data recorded at different times of each individual evolution, did not allow another approach as time-dependant Cox proportional hazards models. The compliance to the Guidelines recommendations on frequency of biochemistry studies (serum calcium, phosphate, or iPTH) were not evaluated, as the NRHP-UY is a voluntary registry and there may be sub-registration that may lead to inaccurate conclusions. Although other authors had found similar results [12], it was surprising that there were iPTH data only in 2013 (31.1%) CKD G3b-4 patients, as Guidelines [7,14] recommended its measure when eGFR <60 mL/min/1.73 m^2^. The shorter follow-up time of No PTH-data group (Table 2) may contribute to explain this.

So, the studied population in Model 2 may have a ‘selection bias’ as there were differences between those with and without PTH-data (Tables 1–3). The PTH-data group were younger, their baseline eGFR were lower, less patients had lower calcium or higher phosphate levels, vitamin D treatment was more frequent, and death incidence rate was lower (Tables 1–3).

**Table 3.** Evolution data.

CKD grade 3b-4	Global	PTH-data	No PTH-data
Number	6473	2013	4460
Death, *n* (%)	2426 (37.5%)	469 (23.3%)	1957 (43.9%)
KRT, *n* (%)	618 (9.5%)	181 (9.0%)	437 (9.8%)
Follow-up time (sum) (patient–year)	38,030	13,850	24,180
Follow-up time (months) (med pc 25–75)	65.5 (33.2–102.1)	81.5 (47.9–113.6)	59.0 (28.2–95.1)
Incidence rate death (events/100 patient–year)	6.38	3.39	8.09
Incidence rate KRT (events/100 patient–year)	1.62	1.31	1.81

KRT: kidney replacement therapy.

Follow-up time, incidence rates (KRT and death).

According to KDIGO 2009 and SEN guidelines 2011 [7,22], this studied population accomplished calcium target (‘normal range’ between 8.4 and 10.0 mg/dl) in 86.9%, phosphate target (between 3 and 4.5 mg/dl) in 78.4%, and mean iPTH 35–105 pg/ml in 848 (42.1%) (Table 2).

The KDIGO guidelines 2017 [9] suggest, in patients with CKD G3a–G5D, that treatment should be started at overt hyperphosphatemia and that a mild and asymptomatic hypocalcemia can be tolerated in order to avoid inappropriate calcium loading. Previous guidelines [5] suggested to maintain serum calcium and phosphate ‘within the normal ranges’ but the 2017 Work Group concluded that: the association between serum phosphate and outcome was not monotonic, the efficacy of diet or phosphate binders in CKD G3-4 was not demonstrated and neither was their safety.

The lower mortality risk ranges observed in the present study (Model 1) were slightly different than those proposed by those guidelines (Supplementary Tables 1–3, Figures 2–4). As it was observed in the PECERA study [13] (although with a different methodology) and by other authors [23,24], U curves were described by the serum calcium and phosphate levels and the mortality risk, in the bivariate, separately, analysis (Figures 2 and 3). However, the PTH curve (Figure 4) showed a linear association, different from that observed by other authors [13,23,24], that may be explained by the ‘selection bias’ previously mentioned. As it was a ‘real world’ study, probably the attending nephrologists did not measure iPTH in older and stable CKD G3b patients, in spite of the Guidelines recommendations, or the data were not registered. Vitamin D insufficiency is more prevalent in CKD than in the general population [25]. It is a modifiable risk factor for secondary hyperparathyroidism that should be corrected in CKD, and supplementation with nutritional vitamin D is recommended by clinical practice guidelines [9]. In 2003, the National Kidney Foundation defined vitamin D sufficiency as serum total 25(OH) vitamin D concentrations of ≥30 ng/mL [8], and in 2011, the Endocrine Society defined it as concentrations between 30 and 100 ng/mL [26]. In the general population, restoring 25(OH) vitamin D levels with nutritional supplementation reduced the risk of mortality [27]. In the present study, serum 25(OH) vitamin D levels ≤ 10 ng/ml were significantly associated with higher mortality (Figure 5, Supplementary Tables 4–6).

The multivariate Cox regression showed, as in previous national studies [15–17,28] that age, diabetes, cardiovascular comorbidities, proteinuria, and GFR were independently associated with higher mortality risk and that RASB was independently associated with a lower mortality risk (Supplementary Table 4). A higher adjusted mortality risk was significantly associated with serum Calcium ≤9, serum phosphate >4 mg/dl, and iPTH >105 pg/ml (Model 2) (Table 4). Treatment with 25(OH) vitamin D was associated with a significant lower mortality risk (Supplementary Table 5).

**Table 4.** (Model 2) Death risk.

Death risk (Model 2)	HR	CI 95%	*p*
Serum calcium (mg/ml)			
9.01–10.25 mg/dl [reference]			.005
≤9.0 mg/dl	1.391	1.127–1.707	.002
>10.25 mg/dl	1.449	0.904–2.322	.123
Serum phosphate (mg/dl)			
2.76–4.0 mg/dl [reference]			.012
≤2.75 mg/dl	1.137	0.616–2.099	.680
>4.00 mg/dl	1.387	1.118–1.720	.003
Serum iPTH (pg/ml) [reference ≤105 pg/ml]			
Serum iPT*H* > 105 pg/ml	1.275	1.049–1.550	.015

iPTH: intact parathyroid hormone.

Cox regression multivariate analysis, adjusted to sex, age, diabetes, smoking, CKD etiologies, CV comorbidities, eGFR, initial systolic and diastolic blood pressure, proteinuria, RASB, 25(OH) and 1,25(OH)_2_ vitamin D treatments, serum calcium, serum phosphate, and serum iPTH (*n* = 2013).

The multivariate Cox regression adjusted also to serum 25(OH) vitamin D levels (Model 3) showed similar results (Supplementary Tables 5 and 6) except that (in this reduced population) low serum calcium levels were associated with a higher mortality (although not significantly) and treatment with 1,25(OH) vitamin D, but not with 25(OH), was associated with a significant lower mortality risk. These results emphasized the importance of including serum 25(OH) vitamin D levels and treatment in the analysis of CKD-MBD [9–11].

The higher mortality risk observed associated with hyperphosphatemia was consistent with previous observations [11,22,29] and with KDIGO guidelines [3,9–11]. Tonelli et al. (30) had found a graded independent relation between higher levels of serum phosphate and cardiovascular events and mortality risk, but COSMOS [10,11] and PECERA [13] studies found a U-shape relation. The present global population showed a U-shape association between serum phosphate and mortality risk in the bivariate Cox regression analysis, but when adjusted to covariates (Models 2 and 3), only serum phosphate higher than 4 mg/dl were significantly associated with higher mortality risk. But in the PTH-data Group there were only 61 patients with mean serum phosphate ≤ 2.75 mg/dl (Table 2) and that may limit statistical analysis. Phosphate control was achieved mainly by dietary protein and processed food restrictions, following national and international guidelines [5,9,14] and as suggested by many authors [3,31–33]. Patients received nutritional advice as NRHP-UY units included Nutritionists, according to the National Guidelines [14]. Phosphate binders use was not registered and those calcium-based were discouraged by the Guidelines and infrequently prescribed [9,14,24]. Melamed et al. [34] remarked that there was more evidence for treating hyperphosphatemia than hyperparathyroidism, but they warned about chelating treatments’ side-effects. Phosphorus excretion was not routinely measured, so there were no data about this important parameter associated with vascular calcification in CKD G3 [35].

Most patients had mean serum calcium ‘on target’, 45.6% received 25(OH) vitamin D and 25.4% received 1,25(OH)_2_. Serum 25(OH) vitamin D (median value of 25.5 (17.0–33.0) ng/ml), was not significantly different between groups with and without PTH data (Table 2). The present global population also showed a U-shape association between serum calcium and mortality risk in the bivariate Cox regression analysis, but when adjusted to covariates (Model 2) only low calcium levels were significantly associated with higher mortality risk and in Model 3, when adjusted also to serum vitamin D levels, neither low nor high calcium levels were significantly associated with higher mortality. The COSMOS and PECERA studies [10–13] as well as Kovesdy et al. [36], observed that high and low serum calcium levels were associated with higher mortality. As in the present study, the multivariate analysis was performed in the PTH-data group there were only 75 patients with mean serum calcium >10.25 mg/dl (Table 2) and that may limit the statistical analysis. Otherwise, Fouque et al. [37] did not observe such association between abnormal serum calcium, phosphate or PTH and all-cause mortality risk in a small French cohort CKD 4-5 patients.

An iPTH >105 pg/ml was associated with higher mortality risk, as referred by other authors [6–9], although in the PECERA study a *U*-shaped association was observed [13]. In the present studied population, only 87 patients had serum iPTH ≤ 35 pg/ml (Supplementary Table 3), so the statistical analysis may be limited. Evenepoel et al. [38] emphasized on parathyroid glands hyporesponsiveness in CKD patients and that optimal PTH range may be define at a population level, but it may be difficult at an individual patient level. KDIGO guidelines [9] suggested that as ‘optimal iPTH level’ are not known, if it raises progressively or persistently above the upper ‘normal limit for the assay’, hyperphosphatemia, hypocalcemia, high phosphate intake, and vitamin D deficiency (‘the modifiable factors’) must be evaluated and eventually treated.

In Model 3, multivariate Cox regression analysis treatment with 1,25(OH) vitamin D was associated with a lower mortality (Supplementary Tables 5 and 6). In a recent meta-analysis [39] that includes 14 observational studies (194,932 dialysis and no-dialysis patients), authors conclude that therapies with 1,25(OH)_2_ vitamin D and analogs were associated with reduced mortality in CKD patients, and particularly in those suffering from secondary hyperparathyroidism [39]. Christodoulou et al. [40] made a systematic meta-analysis of trials on vitamin D treatment and they stated that CKD patients were a ‘high risk’ population for vitamin D deficiency, as observed in this study population, due to their protein-restricted diet and reduced cutaneous synthesis. They concluded that there were still gaps in the evidence-based management of vitamin D treatment, with high heterogeneity. Guidelines recommended vitamin D supplementation similar to the general population [9,14] and 1,25(OH)_2_ vitamin D or analogs, only if serum PTH was persistently and progressively over the upper limit of the assay. In the studied population, as a retrospective ‘real world’ analysis, it was difficult to separate serum calcium and vitamin D level and the treatments patients had received, but treatment with 25(OH) vitamin D was independently associated with a lower mortality risk in Model 2 and 1,25(OH) vitamin D in Model 3 adjusted also to serum vitamin D levels (Tables 4 and 5). A more detailed analysis of the population treated with 25(OH) and/or 1,25(OH)_2_ vitamin D is out of the scope of the present study and will be performed in the near future.

**Table 5.** (Model 3) Death risk.

Death risk (Model 3)	HR	CI 95%	*p*
Serum calcium (mg/ml)			
9.01–10.25 mg/dl [reference]			.132
≤9.0 mg/dl	1.265	0.891–1.794	.188
>10.25 mg/dl	1.944	0.865–4.369	.108
Serum phosphate (mg/dl)			
2.76–4.0 mg/dl [reference]			.009
≤2.75 mg/dl	0.828	0.258–2.649	.750
>4.00 mg/dl	1.668	1.201–2.317	.002
Serum iPTH (pg/ml) [reference ≤105 pg/ml]			
Serum iPT*H* > 105 pg/ml	1.386	1.012–1.898	.042
Serum 25(OH) vitamin D (ng/ml) [reference >10 ng/ml]			
Serum 25(OH) vitamin D ≤10 ng/ml	1.958	1.238–3.098	.004

iPTH: intact parathyroid hormone. Cox regression multivariate analysis, adjusted to sex, age, diabetes, smoking, CKD etiologies, CV comorbidities, eGFR, initial systolic and diastolic blood pressure, proteinuria, RASB, 25(OH) and 1,25(OH)_2_ vitamin D treatments, serum calcium, serum phosphate serum iPTH, and serum 25(OH) vitamin D levels (*n* = 964).

### Study strengths and limitations

This study has limitations: (1) The data registry is nonmandatory, and nephrologists report data at different time intervals, so an ideal, more robust, time-dependent Cox proportional hazards models and penalized splines analysis could not be performed, (2) there may be heterogeneities in structure and function among NRHP-UY teams, as well as differences on the recommended frequency of laboratory tests and clinic visits, (3) laboratory tests were performed at different laboratories departments, (4) treatment prescription may not always follow national/international Guidelines, and (5) the limited availability of PTH and vitamin D data restricted the number of patients data included in the multivariate analysis. All these facts may introduce bias.

The study has several important strengths: (1) This is a large cohort with a long follow-up of more than 10 years, in the context of real-world health care. (2) Data collection was conducted prospectively, on-line, in a format created for that purpose, recorded by the attending nephrologists, (3) Outcome indicators are obtained from national mandatory registries.

## Conclusion

In a CKD G3b-4 cohort with a long follow-up, serum calcium ≤9 (if not adjusted to serum vitamin D), serum phosphate >4 mg/dl, serum iPTH >105 pg/ml and serum 25(OH) vitamin *D* ≤ 10 ng/ml were independently associated with higher all-cause mortality risk. These data may contribute to emphasize the importance of a strict monitorization of these intertwined biomarkers in CKD G3b-4 patients and to precise their target levels’ definition.

## Supplementary Material

Supplemental Material

## Data Availability

Upon request, data set could be available as a Supplemental online archive.
