# Supplementary material for: Mineral and bone disorder and longterm survival in a chronic kidney disease grade 3b-4cohort
Source: Ren Fail. 2022 Aug 10;44(1):1356–67. doi: 10.1080/0886022X.2022.2107543 (PMC9373789; doi:10.1080/0886022X.2022.2107543)
Supplement: Supplemental Material [file IRNF_A_2107543_SM2270.pdf]

**Supplementary Table 1. Serum calcium and death risk. Bivariate Cox regression analysis (n=6473)**

| Serum calcium (mg/dl) | n | HR | CI 95% | p |
|---|---|---|---|---|
| ≤7.50 | 83 | 2.354 | 0.667 - 3.323 | 0.000 |
| 7.51-7.75 | 18 | 2.452 | 1.264 – 4.757 | 0.008 |
| 7.76-8.00 | 51 | 2.969 | 1.990 – 4.430 | 0.000 |
| 8.01-8.25 | 97 | 2.297 | 1.681 – 3.139 | 0.000 |
| 8,26-8.50 | 234 | 2.107 | 1.707 – 2.602 | 0.000 |
| 8.51-8.75 | 362 | 1.936 | 1.610 – 2.327 | 0.000 |
| 8.76-9,00 | 837 | 1.397 | 1.198 – 1.629 | 0.000 |
| 9.01-9.25 | 1118 | 1.156 | 0.998 – 1.340 | 0.053 |
| 9.25-9.50 | 1412 | 1.182 | 1.028 – 1.360 | 0.019 |
| 9.51-9.75 | 998 | Reference | | |
| 9.76-10.00 | 740 | 1.130 | 0.957 – 1.335 | 0.149 |
| 10.01.10.25 | 251 | 0.995 | 0.779 – 1.270 | 0.966 |
| 10.26-10.50 | 126 | 1.390 | 1.039 – 1.859 | 0.027 |
| 10.51-10.75 | 65 | 1.249 | 0.811 – 1.924 | 0.313 |
| 10.76-11.00 | 43 | 2.276 | 1.503 – 3.446 | 0.000 |
| > 11.00 | 38 | 1.874 | 1.192 - 2.944 | 0.006 |
| **Serum calcium (mg/dl)** | **n** | **HR** | **CI 95%** | **p** |
| ≤ 9.00 | 1682 | 1.539 | 1.410 – 1.679 | 0.000 |
| 9.01-10.25 | 4519 | Reference | | |
| > 10.25 mg/dl | 272 | 1.386 | 1.150 – 1.669 | 0.001 |

**Supplementary Table 2. Serum phosphate and death risk. Bivariate Cox regression analysis (n=6473)**

| Serum phosphate (mg/dl) | n | HR | CI 95% | p |
|---|---|---|---|---|
| ≤2.75 | 235 | 1.306 | 1.036 – 1.647 | 0.024 |
| 2.76-3.0 | 454 | 1.076 | 0.893 – 1.295 | 0.441 |
| 3.01-3.25 | 617 | 1.146 | 0.971 – 1,352 | 0.107 |
| 3.26-3.5 | 1063 | 1.127 | 0.978 – 1.298 | 0.098 |
| 3.5-3.75 | 1021 | Reference | | |
| 3.76-4.0 | 1134 | 1.148 | 0.996 – 1.323 | 0.057 |
| 4.01.4.25 | 647 | 1.180 | 1.000 – 1.393 | 0.050 |
| 4.26-4.5 | 534 | 1.445 | 1.219 – 1.713 | 0.000 |
| 4.51-4.75 | 228 | 1.724 | 1.378 – 2.157 | 0.000 |
| 4.75-5.0 | 187 | 1.350 | 1.034 – 1.762 | 0.027 |
| 5.01-5.25 | 76 | 1.699 | 1.177 – 2.452 | 0.005 |
| 5.26-5.5 | 67 | 2.020 | 1.399 – 2.917 | 0.000 |
| >5.5 | 210 | 1.376 | 1.085 – 1.746 | 0.009 |
| **Serum phosphate (mg/dl)** | **n** | **HR** | **CI 95%** | **p** |
| ≤2.75mg/dl | 235 | 1.189 | 0.962 – 1.471 | 0.110 |
| 2.76-4.00 mg/dl | 4289 | Reference | | |
| >4.00 mg/dl | 1949 | 1.263 | 1.158 – 1.377 | 0.000 |

**Supplementary Table 3. Serum parathyroid hormone (iPTH) and death risk. Bivariate Cox regression analysis (n=2013).**

| Serum iPTH (pg/ml) | n | HR | CI 95% | p |
|---|---|---|---|---|
| ≤35.00 | 87 | Reference | | |
| 35.01 a 105.00 | 848 | 1.434 | 0.779 – 2.640 | 0.247 |
| 105.01 a 210.00 | 718 | 1.956 | 1.065 – 3.591 | 0.031 |
| >210.01 | 360 | 2.204 | 1.182 – 4.110 | 0.013 |
| **Serum iPTH (pg/ml)** | **n** | **HR** | **CI 95%** | **p** |
| ≤105.00 | 935 | Reference | | |
| >105.01 | 1078 | 1.456 | 1.208 – 1.756 | 0.000 |

**Supplementary Table 4. Serum 25(OH) vitamin D and death risk. Bivariate Cox regression analysis (n=1228).**

| Serum 25(OH) Vitamin D (ng/ml) | n | HR | CI 95% | p |
|---|---|---|---|---|
| ≤10.00 | 120 | 3.789 | 1.493 – 9.619 | 0.005 |
| 10.01-30.00 | 714 | 1.799 | 0.737 – 4.391 | 0.197 |
| 30.01-50.00 | 350 | 1.662 | 0.670 – 4.124 | 0.273 |
| 50.01-70.00 | 42 | Reference | | |
| >70.01 | 22 | 2.774 | 0.987 – 7.797 | 0.059 |
| **Serum 25(OH)Vitamin D (ng/ml)** | **n** | **HR** | **CI 95%** | **p** |
| ≤10.00 | 120 | 2.169 | 1.541 – 3.051 | <0.001 |
| >10.01 | 1108 | Reference | | |

**Supplementary Table 5 (Model 2). Death risk**. Cox regression multivariate analysis, adjusted to sex, age, diabetes, smoking, CKD etiologies, CV comorbidities, eGFR, initial systolic and diastolic blood pressure, proteinuria, RASB, 25(OH) and $1,25(OH)_2$ vitamin D treatments, serum calcium, serum phosphate and serum iPTH (n=2013).

| **Death risk (Model 2)** | **HR** | **CI 95%** | **p** |
|---|---|---|---|
| Sex [Reference Men] | 0.855 | 0.698-1.046 | 0.128 |
| Age (year) [Continuous] | 1.080 | 1.067-1.094 | <0.001 |
| Diabetes [Reference No] | 1.502 | 1.219-1.851 | <0.001 |
| CKD etiologies | | | |
| Glomerulopathies [Reference] | | | 0.089 |
| Diabetic Nephropathy | 2.859 | 0.871-9.382 | 0.083 |
| Tubulo-interstitial Nephropathy | 3.922 | 1.155-13.320 | 0.028 |
| Vascular | 4.036 | 1.266-12.861 | 0.018 |
| Others | 3.786 | 1.184-12.104 | 0.025 |
| Cardiovascular comorbidities [Reference No] | 1.328 | 1.090-1.619 | 0.005 |
| Smoking [Reference No] | 1.153 | 0.765-1.737 | 0.495 |
| Proteinuria (mg/day) [Reference <300 mg/day] | 1.688 | 1.249-2.280 | 0.001 |
| eGFR (ml/min/1.73m$^2$) [Continuous] | 0.989 | 0.977-1.001 | 0.079 |
| Systolic blood pressure (mmHg) | | | |
| 120-139 mmHg [Reference] | | | 0.297 |
| < 120 mmHg | 0.932 | 0.707-1.229 | 0.619 |
| 140-159 mmHg | 0.794 | 0.623-1.010 | 0.061 |
| ≥ 160 mmHg | 0.838 | 0.602-1.167 | 0.296 |
| Diastolic blood pressure (mmHg) | | | |
| 80-89 mmHg [Reference] | | | 0.930 |
| < 80 mmHg | 0.991 | 0.789-1.245 | 0.940 |
| 90-99 mmHg | 0.894 | 0.629-1.270 | 0.532 |
| ≥ 100 mmHg | 1.009 | 0.594-1.714 | 0.974 |
| RASB treatment [Reference No] | 0.749 | 0.608-0.922 | 0.006 |
| 25 (OH) Vitamin D treatment [Reference No] | 0.704 | 0.575-0.861 | 0.001 |
| 1,25(OH) Vitamin D treatment [Reference No] | 0.864 | 0.675-1.105 | 0.245 |
| **Serum calcium (mg/ml) 9.01-10.25 mg/dl [Reference]** | | | **0.005** |
| **≤9.0 mg/dl** | **1.391** | **1.127-1.707** | **0.002** |
| **> 10.25 mg/dl** | **1.449** | **0.904-2.322** | **0.123** |
| **Serum phosphate (mg/dl) 2.76-4.0 mg/dl [Reference]** | | | **0.012** |
| **≤ 2.75 mg/dl** | **1.137** | **0.616-2.099** | **0.680** |
| **>4.00 mg/dl** | **1.387** | **1.118-1.720** | **0.003** |
| **Serum iPTH (pg/ml) [Reference ≤ 105 pg/ml]** | | | |
| **Serum iPTH >105 pg/ml** | **1.275** | **1.049-1-550** | **0.015** |

eGFR= estimated glomerular filtration rate, iPTH= intact Parathyroid hormone, RASB= renin-angiotensin system blockers.

**Supplementary Table 6 (Model 3). Death risk**. Cox regression multivariate analysis, adjusted to sex, age, diabetes, smoking, CKD etiologies, CV comorbidities, eGFR, initial systolic and diastolic blood pressure, proteinuria, RASB, 25(OH) and $1,25(OH)_2$ vitamin D treatments, serum calcium, serum phosphate, serum iPTH and serum 25(OH)Vitamin D (n=964).

| **Death risk (Model 3)** | **HR** | **CI 95%** | **p** |
|---|---|---|---|
| Sex [Reference Men] | 0.771 | 0.560 – 1.060 | 0.110 |
| Age (year) [Continuous] | 1.066 | 1.047 – 1.086 | < 0.001 |
| Diabetes [Reference No] | 1.457 | 1.033 – 2.054 | 0.032 |
| CKD etiologies | | | |
| Glomerulopathies [Reference] | | | 0.734 |
| Diabetic Nephropathy | 1.958 | 0.564 – 6.795 | 0.290 |
| Tubulo-interstitial Nephropathy | 1.357 | 0.332 – 5.535 | 0.671 |
| Vascular | 1.815 | 0.541 – 6.093 | 0.335 |
| Others | 1.962 | 0.583 – 6.601 | 0.276 |
| Cardiovascular comorbidities [Reference No] | | | |
| Smoking [Reference No] | 0.699 | 0.342 – 1.428 | 0.326 |
| Proteinuria (mg/day) [Reference <300 mg/day] | 1.744 | 1.085 – 2.804 | 0.022 |
| eGFR (ml/min/1.73m$^2$) [Continuous] | 0.979 | 0.959 - 0.998 | 0.032 |
| Systolic blood pressure (mmHg) | | | |
| 120-139 mmHg [Reference] | | | 0.177 |
| < 120 mmHg | 0.688 | 0.446 – 1.061 | 0.091 |
| 140-159 mmHg | 0.583 | 0.348 – 0.977 | 0.040 |
| ≥ 160 mmHg | 0.535 | 0.286 – 1.001 | 0.050 |
| Diastolic blood pressure (mmHg) | | | |
| 80-89 mmHg [Reference] | | | 0.692 |
| < 80 mmHg | 1.210 | 0.823 – 1.780 | 0.331 |
| 90-99 mmHg | 1.337 | 0.784 – 2.280 | 0.286 |
| ≥ 100 mmHg | 1.235 | 0.562 – 2.713 | 0.599 |
| RASB treatment [Reference No] | 0.763 | 0.542 – 1.074 | 0.121 |
| 25 (OH) Vitamin D treatment [Reference No] | 0.946 | 0.676 – 1.326 | 0.749 |
| 1,25(OH) Vitamin D treatment [Reference No] | 0.639 | 0.451 – 0.906 | 0.012 |
| **Serum calcium (mg/ml) 9.01-10.25 mg/dl [Reference]** | | | **0.132** |
| **≤9.0 mg/dl** | **1.265** | **0.891 – 1.794** | **0.188** |
| **> 10.25 mg/dl** | **1.944** | **0.865 – 4.369** | **0.108** |
| **Serum phosphate (mg/dl) 2.76-4.0 mg/dl [Reference]** | | | **0.009** |
| **≤ 2.75 mg/dl** | **0.828** | **0.258 – 2.649** | **0.750** |
| **>4.00 mg/dl** | **1.668** | **1.201 – 2.317** | **0.002** |
| **Serum iPTH (pg/ml) [Reference ≤ 105 pg/ml]** | | | |
| **Serum iPTH > 105 pg/ml** | **1.386** | **1.012 – 1.898** | **0.042** |
| **Serum 25(OH)Vitamin D (ng/ml) [Reference > 10 ng/ml]** | | | |
| **Serum 25(OH)Vitamin D ≤10 ng/ml** | **1.958** | **1.238 – 3.098** | **0.004** |

eGFR= estimated glomerular filtration rate, iPTH= intact Parathyroid hormone,
RASB= renin-angiotensin system blockers.
